# Ultrafast Photoconversion Dynamics of the Knotless Phytochrome *Syn*Cph2

**DOI:** 10.3390/ijms221910690

**Published:** 2021-10-02

**Authors:** Tobias Fischer, Luuk J. G. W. van Wilderen, Petra Gnau, Jens Bredenbeck, Lars-Oliver Essen, Josef Wachtveitl, Chavdar Slavov

**Affiliations:** 1 Institute of Physical and Theoretical Chemistry, Goethe University Frankfurt am Main, Max-von-Laue Straße 7, 60438 Frankfurt, Germany; fischer@theochem.uni-frankfurt.de; 2 Institute of Biophysics, Goethe University Frankfurt am Main, Max-von-Laue Straße 1, 60438 Frankfurt, Germany; vanwilderen@biophysik.uni-frankfurt.de (L.J.G.W.v.W.); bredenbeck@biophysik.uni-frankfurt.de (J.B.); 3 Department of Chemistry, Philipps-Universität Marburg, Hans-Meerwein-Straße 4, 35032 Marburg, Germany; gnau@staff.uni-marburg.de (P.G.); essen@chemie.uni-marburg.de (L.-O.E.); 4 Center for Synthetic Microbiology, Philipps-Universität Marburg, Hans-Meerwein-Straße 6, 35032 Marburg, Germany

**Keywords:** tetrapyrrole-binding photoreceptors, phytochromes, photochemistry, photoisomerization, time-resolved spectroscopy

## Abstract

The family of phytochrome photoreceptors contains proteins with different domain architectures and spectral properties. Knotless phytochromes are one of the three main subgroups classified by their distinct lack of the PAS domain in their photosensory core module, which is in contrast to the canonical PAS-GAF-PHY array. Despite intensive research on the ultrafast photodynamics of phytochromes, little is known about the primary kinetics in knotless phytochromes. Here, we present the ultrafast P_r_ ⇆ P_fr_ photodynamics of *Syn*Cph2, the best-known knotless phytochrome. Our results show that the excited state lifetime of P_r_* (~200 ps) is similar to bacteriophytochromes, but much longer than in most canonical phytochromes. We assign the slow P_r_* kinetics to relaxation processes of the chromophore-binding pocket that controls the bilin chromophore’s isomerization step. The P_fr_ photoconversion dynamics starts with a faster excited state relaxation than in canonical phytochromes, but, despite the differences in the respective domain architectures, proceeds via similar ground state intermediate steps up to Meta-F. Based on our observations, we propose that the kinetic features and overall dynamics of the ultrafast photoreaction are determined to a great extent by the geometrical context (i.e., available space and flexibility) within the binding pocket, while the general reaction steps following the photoexcitation are most likely conserved among the red/far-red phytochromes.

## 1. Introduction

Phytochromes represent a superfamily of photosensory receptors that regulate photomorphogenesis, photoprotection, phototaxis and other biologically relevant processes in a variety of organisms [1,2]. Their function is enabled by the Z ↔ E photoisomerization of a bilin chromophore embedded in the protein scaffold. The isomerization is followed by several intermediate steps involving reorganization of the protein matrix. Ultimately, these transformations result in photoswitching between a dark-adapted and a light-adapted state. Recently, phytochromes have attracted considerable attention due to their application potential in optogenetics and as fluorescent probes in biomedical imaging [3,4,5,6].

**Figure 1:**
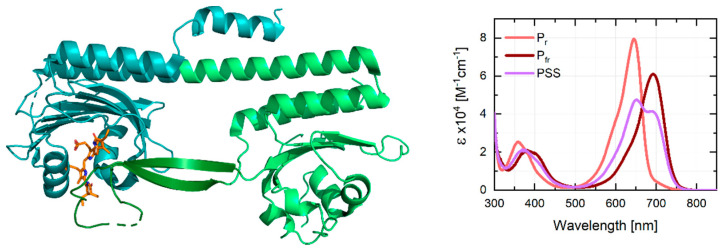
Left: Schematic representation of the structure of *Syn*Cph2 (PDB ID: 4BWI) [51]. The phycocyanobilin (PCB) chromophore (orange) is embedded in the GAF domain (cyan) interacting with a tongue-like protrusion (dark green) of the PHY domain (light green). Right: Absorption spectra of *Syn*Cph2 in the P_r_ (light red) and P_fr_ (dark red) state. To obtain the pure P_fr_ spectrum 36% of the pure P_r_ spectrum (factor determined by visual inspection of the resulting spectrum) was subtracted from the photostationary state (PSS) spectrum (purple), followed by multiplication with a factor of 1.56 to simulate full conversion to the P_fr_ state (as done previously [50]). Fluorescence spectra of the P_r_ state and a fluorescence quantum yield of 3.2% were reported previously while no fluorescence was observed for the P_fr_ state [13].

Phytochromes can be classified into subgroups depending on the domain architecture of their photosensory core modules (PSM). In group I phytochromes, comprised of canonical plant and cyanobacterial phytochromes (e.g., PhyA and *Syn*Cph1) as well as bacteriophytochromes (e.g., Agp1 and DrBphP), the PSM consists of a PAS-GAF-PHY array where all three domains are necessary for photoconversion [7,8,9]. The PAS domain forms a figure-of-eight knot structure with the chromophore-binding GAF domain, whereas the phytochrome specific PHY domain interacts with the chromophore in the GAF domain via a tongue like protrusion which stabilizes the chromophore and shields it from the solvent [1,2,10,11]. Group II phytochromes, also termed knotless or Cph2-like phytochromes, lack the PAS domain (Figure 1) and mostly consist of cyanobacterial phytochromes with Cph2 from *Synechocystis* sp. (*Syn*Cph2) being the most prominent member [1,2,12,13]. Lastly, Group III consists of cyanobacteriochromes (CBCRs) where single GAF domains form the PSM [14]. While the phytochromes of Group I and II photoconvert almost exclusively between P_r_ (red-absorbing) and P_fr_ (far-red-absorbing) states, CBCRs exhibit significant spectral diversity covering the near-UV/visible/near-IR spectral range [13,15,16,17,18,19,20,21,22].

The primary photoconversion dynamics of phytochromes is highly complex, often involving ground state heterogeneity and/or multiple excited state decay pathways. The P_r_ photoisomerization kinetics varies significantly amongst the phytochromes from Groups I and III. In canonical phytochromes (e.g., PhyA, *Syn*Cph1, Agp1), the kinetics can be described by three processes: (i) ultrafast departure from the Franck-Condon region (sub-ps lifetime); (ii) conformational dynamics of the chromophore with the onset of D-ring rotation (~2 to 5 ps lifetime) and (iii) formation of the primary red-shifted photointermediate Lumi-R (~30 to 50 ps lifetime) [23,24,25,26,27,28,29]. Alternatively, processes (ii) and (iii) have also been interpreted as kinetics of two different populations in the context of ground state heterogeneity [23,30,31]. In bacteriophytochromes, overall longer excited state lifetimes (~100 to 300 ps) have been observed which were linked to excited state proton transfer and reorganization of the hydrogen bonding network [32,33,34,35,36,37]. The excited state kinetics is prolonged to hundreds of picoseconds also in CBCRs. In some studies the kinetics was modelled by a series of non-reactive and reactive populations based on ground state heterogeneity [38,39,40], while other works hint towards active site relaxation and distributed type kinetics rather than distinct heterogeneity [41,42,43].

Presently, the primary photoisomerization in the reverse reaction (P_fr_ → P_r_) is exclusively studied in Group I phytochromes. It is relatively conserved amongst the different representatives of the group and occurs faster than the forward (P_r_ → P_fr_) reaction. Following the ultrafast departure from the FC-region (~100 fs), the primary photointermediate Lumi-F is formed on the sub-ps time scale (~500 to 700 fs lifetime) [27,28,44,45,46,47,48]. This intermediate evolves on the ps time scale via several relaxation steps resulting in a spectral blue shift [27,45,47]. In *Syn*Cph1, multiple excited state decays have been proposed and were linked to reactive and non-reactive chromophore conformations with different lifetimes [45,47]. Similar observations in the bacteriophytochromes PaBphP (bathy) and Agp1 were instead interpreted as branching on the excited state surface [48,49]. Such multiphasic kinetics was not reported in oatPhyA, where a single excited state decay was sufficient to describe the data [27].

In contrast, little is known about the ultrafast forward and reverse dynamics of Group II phytochromes. To date, only the forward photoisomerization direction of the knotless phytochrome All2699g1g2 has been examined [50], but no details on the reverse reaction are available. To shed light on the ultrafast primary photoreaction of knotless phytochromes, here we present both the ultrafast forward (P_r_ → P_fr_) and reverse (P_fr_ → P_r_) dynamics of the most prominent group member, *Syn*Cph2.

## 2. Results and Discussion

### 2.1. Ultrafast P_r_* Dynamics and Lumi-R Formation

**Figure 2:**
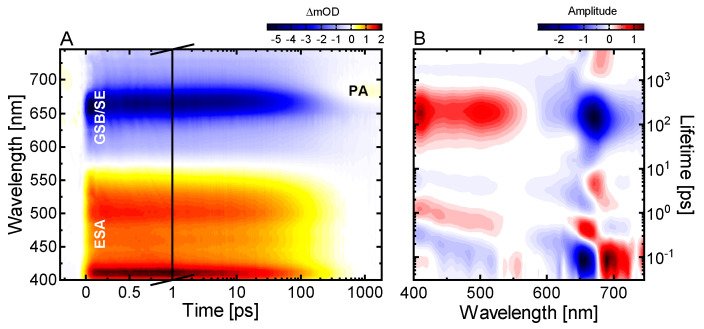
Ultrafast excited state dynamics of the P_r_ state in *Syn*Cph2 investigated in the vis spectral range. (**A**) TA data of the P_r_ → P_fr_ dynamics after excitation at 640 nm. (**B**) Corresponding lifetime density map (LDM) obtained from the lifetime distribution analysis of the TA data displayed in (**A**). The GSB signal coincides with the ground state absorption spectrum in Figure 1.

The femtosecond transient absorption (TA) data show three major contributions (Figure 2A): (i) a broad positive signal below ~550 nm due to excited state absorption (ESA), (ii) a broad negative signal extending from 575 nm to 740 nm due to P_r_ ground state bleach (GSB) and stimulated emission (SE) and (iii) a positive signal (product absorption; PA) at 675 nm which is uncovered after the decay of SE and the partial recovery of the GSB and is accordingly assigned to the absorption of the primary photoproduct Lumi-R.

We performed lifetime density analysis (LDA) (for details see Material and Methods or [52]) to reveal further features of the excited state dynamics (Figure 2B). The negative- and positive-amplitude lifetime distributions at ~100 fs from 600 nm to 675 nm and 675 nm to 740 nm respectively, represent the red shift of the SE signal and can be assigned to the departure from the FC-region. The following pairs of negative- and positive-amplitude distributions at ~0.6 ps and 2–8 ps show slight spectral changes at the edge of the GSB and SE signals. These can be ascribed to changes on the excited state surface related to conformational dynamics of the chromophore. The dominant feature of the lifetime density maps (LDM) is located at ~200 ps with a positive-amplitude distribution in the range of the ESA signal (400–575 nm) and a negative-amplitude distribution in the GSB-SE range (600–740 nm) indicating the decay of the excited state, the recovery of the ground state and the formation of the primary photoproduct Lumi-R. The broadness of the lifetime distributions reflects the distributed type of the excited state decay kinetics as shown by the detailed analysis in our previous studies [50,53]. The final pair of lifetime distribution amplitudes at 3 ns represents the remaining GSB and Lumi-R absorption at 680 nm.

**Figure 3:**
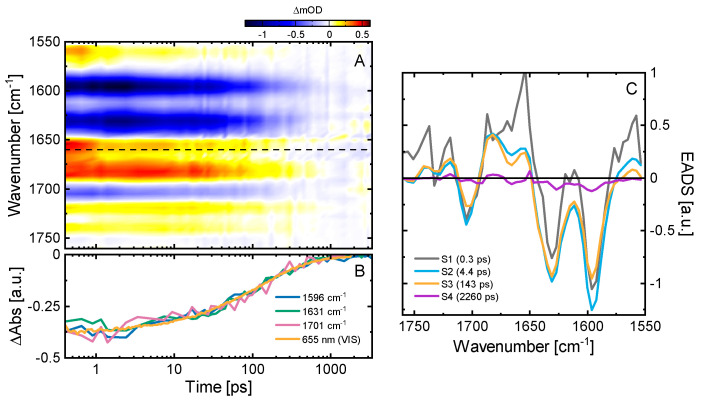
Ultrafast excited state dynamics of the P_r_ state in *Syn*Cph2 investigated in the IR spectral range. (**A**) TA data of the P_r_ → P_fr_ dynamics after excitation at 640 nm. The signal sizes in the C=O region were multiplied by a factor of 2 to account for the lower excitation density used as compared to the data of the C=C region. The transition between these regions is indicated by the dashed black line. (**B**) Scaled transients at selected wavenumbers in comparison to a transient from the GSB of the corresponding vis pump-probe measurements (Figure 2A). (**C**) Evolution-associated difference spectra (EADS) obtained from a sequential model fit of the transient vis pump-IR probe data of the P_r_ dynamics.

Additionally, we performed ultrafast vis pump-IR probe TA experiments covering two regions of interest comprising predominantly the C=O (1750–1670 cm^−1^) and the C=C (1670–1550 cm^−1^) vibrations of the bilin chromophore (Figure 3A). In the C=O region, we identify three major contributions: a negative signal at 1705 cm^−1^, and two positive features at 1745–1715 cm^−1^ and 1682 cm^−1^. Based on previous reports [23,29,54], we assign the 1705(−)/1682(+) signal pair to the GSB and ESA signals of the C_19_=O stretch vibration at ring-D, and the 1745–1715 cm^−1^ positive features to the C_1_=O stretch vibration at ring-A. The corresponding C_1_=O negative GSB signal is plausibly weaker and narrower than the positive ESA band, and thus is responsible for the dip in the 1745–1715 cm^−1^ band. The two prominent negative signals at 1596 and 1630 cm^−1^ and the minimum at 1665 cm^−1^ are related to C=C stretch vibrations of the chromophore. The comparison with previous studies on *Syn*Cph1 and oatPhyA suggests that the 1630 cm^−1^ signal originates from the C_15_=C_16_ mode, while the 1596 cm^−1^ signal is either due to the C_9_=C_10_ mode or to a delocalized mode containing this vibration [23,54,55]. The positive signal at 1560 cm^−1^, the maximum between the two negative signals at 1596 and 1630 cm^−1^, and the positive signal at 1654 cm^−1^ represent the respective ESAs of these vibrations which are all short lived and downshifted due to a reduction of the bond order [54,56].

Our global target analysis (for details see Material and Methods or [52,57,58]) of the IR TA data using a sequential model resulted in four kinetic components (Figure 3C). The first lifetime component (300 fs) is at the limit of our experimental time resolution, and while straightforward assignment is hard, it is plausibly related to early dynamics on the excited state potential energy surface as indicated by the vis TA data (see above). The 4.4 ps lifetime describes a slight decrease of the GSB of the C_9_=C_10_ or delocalized mode, its corresponding ESA and the C_19_=O GSB, while most other signals remain unaffected. The lack of simultaneous decay of all ESA signatures indicates that this lifetime component is associated with excited state conformational changes within the chromophore. This result agrees well with the vis TA data and with our conclusion that (1) only excited state chromophore dynamics occurs on the sub-50 ps timescale and (2) that the P_r_^*^ kinetics of knotless phytochromes does not involve an early excited state decay. Here, the excited state decay occurs with a 143 ps lifetime as designated by the disappearance of the excited state signatures in the evolution-associated difference spectra (EADS) going from S3 to S4 (Figure 3C). The 143 ps lifetime from the IR TA data (Figure 3A) matches the position of the broad lifetime distribution from the visible TA data (Figure 2B). This remarkable kinetic similarity between the UV/vis and the IR data of P_r_*, despite the use of D_2_O buffer for the IR experiments, is also readily observed by direct comparison of the scaled GSB transient decays (Figure 3B). The final EADS corresponds to the IR difference spectrum of the primary photoproduct Lumi-R. The EADS shows remaining GSB signals in the C=C region, while in the C=O region the signals are smaller than what we can resolve at the experimental S/N ratio. The ratio of the GSB amplitudes of the EADSs of S2 and S4 indicates a quantum yield of ~11% for Lumi-R formation, which is in excellent agreement with the ~12% determined previously [13].

### 2.2. Conserved P_r_ Kinetics

**Figure 4:**
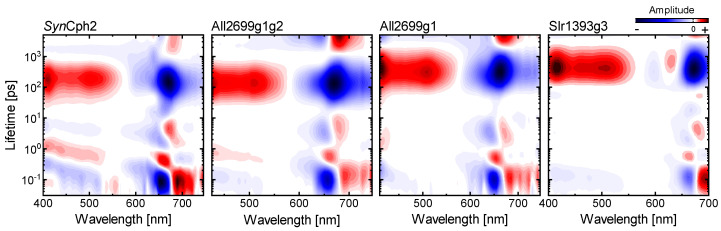
Lifetime density maps of several longer lived phytochromes with similar kinetics including *Syn*Cph2, All2699g1g2 [50], All2699g1 [53] and Slr1393g3 [41,59].

LDMs can be viewed as a kinetic footprint of a given system. In this regard, we noticed that the LDMs describing the P_r_ kinetics in the single GAF domain All2699g1 of a knotless phytochrome [53], the complete PSMs of two knotless phytochromes (All2699g1g2 [50], *Syn*Cph2 (Figure 2)) and the red-green CBCR Slr1393g3 [41] show a striking level of similarity despite the structural and evolutionary differences of these phytochromes (Figure 4). The LDMs, as well as the transient data, of all these longer-lived phytochromes exhibit a common pattern of a sub-200 fs departure from the FC-region followed by two pairs of lifetime distributions at ~0.6 ps and 2–8 ps and a distributed excited state decay in the hundreds of ps time range. Notably, of this pattern only the distributed decay varies in lifetime and is shifted independently of the first two features. Overall, the similarity hints towards a common mechanistic basis for the P_r_ kinetics in these phytochromes.

As we previously concluded based on theoretical calculations and kinetic modelling, the origin of the distributed kinetics can be linked to the protein environment being the controlling factor for excited state relaxation [50,53]. As it constrains the bilin chromophore, and in particular the D-ring, the reaction cannot proceed unless the protein binding pocket undergoes rearrangements that facilitate chromophore isomerization. Potentially, the pocket rearrangements occurring on timescales >50 ps are triggered by slight conformational changes within the chromophore as deduced by the 0.6 ps and 2–8 ps lifetimes from both the vis and IR TA data. Our conclusions are in agreement with other ultrafast studies on the P_r_ state of a bacteriophytochrome and two CBCRs, where the authors identified active site relaxation of the protein matrix and solvation as the cause of the extended multiphasic kinetics instead of ground state heterogeneity [36,42,43]. Based on the experimental data similarities, we expect that the P_r_ forms in the latter studies would yield very similar LDMs as the ones shown in Figure 4.

In contrast, the P_r_ forms of other phytochromes, such as canonical phytochromes (e.g., Agp1, *Syn*Cph1 and oatPhyA) [23,24,27,28,29,31] and some CBCRs [38,40] exhibit multiphasic excited state decays with significantly shorter lifetimes in the range of a few ps and 30 to 50 ps. The occurrence of multiphasic P_r_ behavior has often been linked to distinctly different P_r_ ground states [23,30,31,39]. The binding-pocket-relaxation model that we have proposed for the dynamics of the slower P_r_ forms (e.g., the phytochromes shown in Figure 4) does not explicitly exclude this type of heterogeneity as a contributing kinetic factor. However, we do not observe distinctly heterogeneous dynamics in terms of evolution of separable substates, instead some ground state heterogeneity might add to the distributed character of the dynamics. Therefore, we believe that for the longer-lived phytochromes (e.g., knotless phytochromes [50,53], some bacteriophytochromes [35,36,56] and some CBCRs [40,41,42,43]) the reorganization of the binding pocket after photoexcitation plays a dominant role in determining the isomerization kinetics.

In this regard, the available space and the flexibility of the chromophore-binding pocket, especially around the D-ring, have been proposed as the main factors to explain the extended P_r_ kinetics [36,42,50,53]. Mutational studies have identified highly conserved key amino acids (e.g., Y133, Y47, D79 and H160 in *Syn*Cph2 or Y263, Y176, D207 and H290 in *Syn*Cph1 and DrBphP) in the immediate vicinity of the D-ring that directly affect the excited state lifetime and the isomerization quantum yield [10,13,36,42,60,61,62,63]. In case of the Tyr residues both their steric interactions with the chromophore as well as their participation in the hydrogen bonding network have been proposed as essential for efficient photochemistry [60,62,63,64,65]. Additionally, solvent exposure, the overall hydrogen bonding network and the protonation state of several residues inside the pocket also appear to be important for its properties [34,36,42,43,60,61,66] and the ensuing kinetics.

The concept of pocket flexibility does not contradict the observations made for phytochromes with shorter P_r_ decay lifetimes (e.g., Agp1, *Syn*Cph1, oatPhyA, NpR6012g4). Here, the pocket would have to be more flexible and spacious to allow faster relaxation with less steric hindrances. This higher flexibility could also allow for alternative configurations of amino acid side chains and the chromophore itself resulting in the suggested ground state heterogeneity and the distinct kinetics of substates. In essence, the overall rather similar kinetics of phytochromes, despite significant structural differences, can be rationalized well by the binding-pocket-relaxation model when flexibility is considered.

### 2.3. Ultrafast Dynamics of P_fr_*

**Figure 5:**
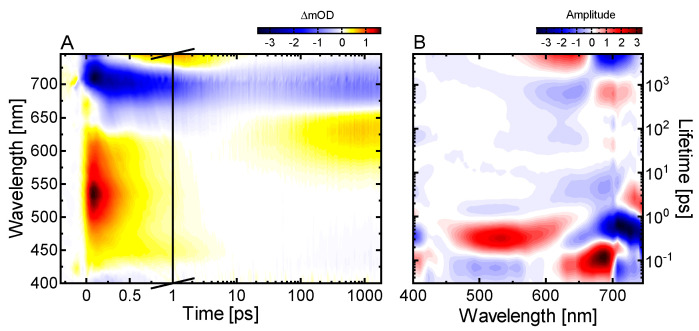
Ultrafast excited state dynamics of the P_fr_ state in *Syn*Cph2 investigated in the UV/vis spectral range. (**A**) TA data of the P_fr_ → P_r_ dynamics after excitation at 710 nm. (**B**) Corresponding lifetime density map (LDM) obtained from the lifetime distribution analysis of the TA data displayed in A).

The femtosecond TA experiments on the primary reverse dynamics of *Syn*Cph2 show four main contributions (Figure 5A). A broad ESA (positive) signal is present below 625 nm, while the GSB and SE (negative) signals extend from 640 nm to 750 nm. At the long wavelength side of our spectral window (730–750 nm), a narrow positive signal appears after ~0.5 ps concomitantly with the decay of the ESA and SE signals. This new absorption signal is assigned to a ground state intermediate (GSI). At longer delay times, positive absorption (550–650 nm) is built up extending to the end of the experimentally accessible time range. These signals can be assigned to later ground state intermediates.

The details of the underlying kinetics are revealed by the corresponding LDM (Figure 5B). The negative- (450–600 nm, 700–750 nm) and positive-amplitude distributions (610–700 nm) at ~100 fs represent the spectral shifts of the ESA and the SE associated with the departure of the excited state wavepacket from the FC-region. The following positive-amplitude distribution at ~300 fs from 450 nm to 650 nm marks the decay of the ESA, while the simultaneous SE decay and partial ground state recovery are accounted for by the negative-amplitude distribution between 670 nm and 750 nm. The extension of the strong negative amplitude to the red is due to the rise of the positive signal of the first GSI located at ~740 nm. In the 1–5 ps lifetime range, the positive- (730 nm to 750 nm) and the negative-amplitude (600 to 700 nm) distributions show the decay of this first GSI to form the next, blue-shifted GSI and to partially recover the GSB. This is clearly visible in the TA data as a decrease in GSB amplitude. The following blue spectral shift evolution over several GSIs is accounted for by the positive- (675–740 nm) and negative- (550–670 nm) amplitude distributions between 50 ps and 1000 ps. On the ns timescale, the last negative- and positive-amplitude lifetime distribution corresponds to the residual signal of the last detectable (on this timescale) GSI and the remaining GSB signal.

**Figure 6:**
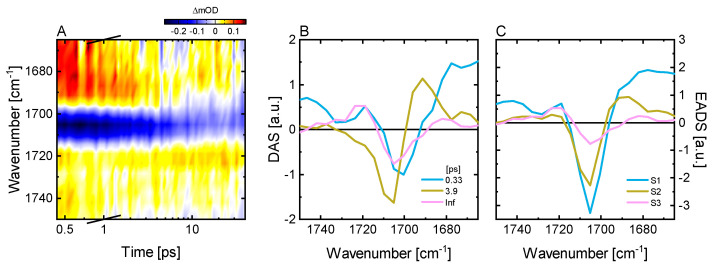
Ultrafast excited state dynamics of the P_fr_ state in *Syn*Cph2 investigated in the IR spectral range. (**A**) TA data of the P_fr_ → P_r_ dynamics after excitation at 710 nm. (**B**) Corresponding decay associated spectra and (**C**) evolution-associated difference spectra (EADS) obtained from a sequential model fit of the data displayed in (**A**).

The vis pump-probe results are further corroborated by our vis pump-IR probe measurements in the C=O region (Figure 6A). The negative GSB of the C_19_=O vibration is located at 1705 cm^−1^ while its corresponding broad ESA is located between 1695 cm^−1^ and 1660 cm^−1^. The positive signal in the 1750–1715 cm^−1^ range with a dip at 1730 cm^−1^ can be assigned to the broad ESA of the C_1_=O and its underlying narrow GSB, respectively. These assignments are based on previous reports on the P_fr_ state of other phytochromes [45,46,48,67]. The target analysis of the data using a sequential model reveals that the ESAs decay with a lifetime of ~330 fs (Figure 6B,C). This decay is accompanied by the rise of a new positive contribution at 1690 cm^−1^ which can be linked to the formation of a GSI, as indicated also by the vis TA data (see above). In a next step, this intermediate decays with a 3.9 ps lifetime which leads to a recovery of most of the GSB and concurrent formation of a positive signal at 1720 cm^−1^. According to the assignment for *Syn*Cph1 [45,46], the 1720 cm^−1^ positive band can be attributed to the Lumi-F intermediate. We observe no further changes in the C=O range in our measurements (up to 40 ps).

### 2.4. Kinetic Model of the Primary P_fr_ Kinetics

Based on the qualitative insight provided by the LDA of the vis data, we constructed kinetic models and fitted those directly to the experimental data to obtain quantitative information (kinetic rates for the different transition steps and the spectra of the involved species) on the P_fr_ kinetics (see refs [52,57,58] for details on global target analysis and the Methods section for specific technical details). The P_fr_ LDM (Figure 5B) clearly shows only one lifetime distribution (at about 300–400 fs) accounting for the decay of the relaxed excited state. Consequently, we used homogeneous kinetic models in the global target analysis. We added a FC state and several GSI states to account for the kinetic features in the LDM. In a next step, we tested models with different connectivity patterns between the states. To support the selection of the most adequate model, we introduced the overall quantum yield of the productive relaxation channel as an additional constraint. This was set to 0.2 as reported previously [13].

**Figure 7:**
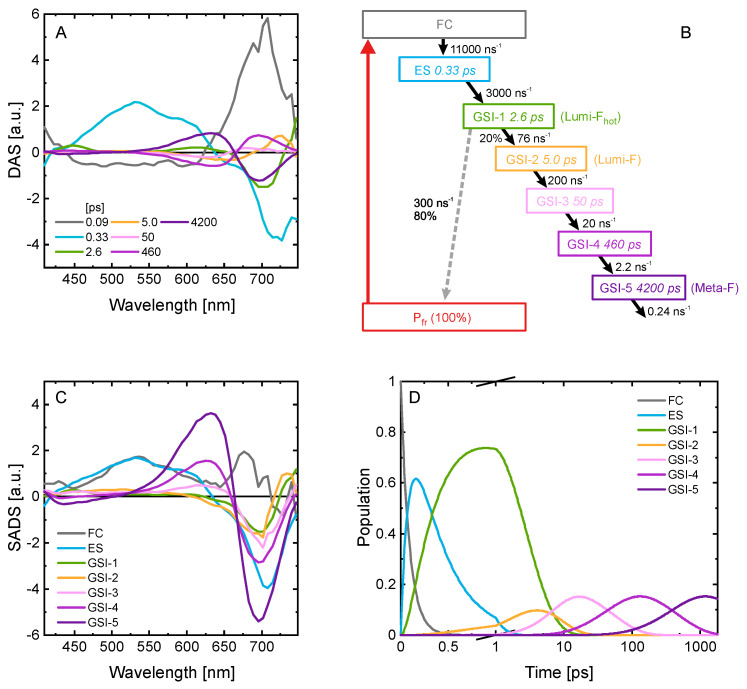
Decay-associated spectra (DAS) (**A**), species-associated difference spectra (SADS) (**C**) and populations (**D**) of individual states obtained from the GTA on the TA data of P_fr_*. The assignment to excited state or GS dynamics is based on the presence of ESA in the 410 to 600 nm range. (**B**) Schematic representation of the best kinetic model showing the rates (in ns^−1^), the decay lifetimes (in ps), and the efficiencies of the corresponding reactions (in percent). Colors match the respective DAS, SADS and populations. The overall QY of the productive pathway was set to 0.2.

Our analysis resulted in a model with physically reasonable kinetic rates and species-associated difference spectra (SADS) (Figure 7). The model shows that the excited state relaxation occurs with a ~300 fs lifetime and leads to the formation of the first ground state intermediate (GSI-1). The SADS of GSI-1 has an absorption peak at about 750 nm (at the edge of our spectral detection window). This state then relaxes with an overall lifetime of 2.6 ps either back to the original P_fr_ ground state (80% yield) or forms GSI-2 (~730 nm absorption, 20% yield). The transition of GSI-1 to GSI-2 is also reflected in the 1–4 ps blue spectral shift dynamics captured by the LDM (Figure 5B) where the positive-amplitude (red) lifetime distribution (710–750 nm) accounts for the decay of the initially formed GSI-1. As indicated by the tilted shape of this distribution the dynamics is non-exponential and involves cooling towards either the P_fr_ ground state or the GSI-2. In turn, GSI-2 decays with about 5 ps lifetime. Note, the 3.9 ps lifetime recovered from the IR TA data (Figure 6) appears as a mixture of the GSI-1 and GSI-2 lifetimes, which can be attributed to the low signal-to-noise ratio of the IR data that does not permit resolving both lifetimes. Next, in a series of steps GSI-2 converts towards GSI-5 (Figure 7A,B), which is the final intermediate that we can observe on our detection timescale (i.e., 1.8 ns). The transition is associated with a gradual blue shift of the SADS of the intermediates, first to GSI-3 at ~690 nm (strongly overlapping with the GSB), consecutively to GSI-4 at 645 nm, and finally to GSI-5 with a maximum at 640 nm (Figure 7C).

In addition to the model in Figure 7, we also tested several other models that included either direct excited state relaxation to the P_fr_ ground state or a P_fr_ hot ground state (see SI, Figures S4–S6). In the models with P_fr_ hot ground state, the SADS with absorption at 750 nm represent the hot ground state. In addition, the blue side of the corresponding SADS is very similar to the SADS of some of the later intermediates and/or the GSI-1 SADS shows a relatively broad GSB contribution. These spectral features are unreasonable; thus we clearly favor the model shown in Figure 7.

### 2.5. Comparison to Other Phytochromes

The primary P_fr_ kinetics of *Syn*Cph2 is very similar to what is reported for other phytochromes. After a fast relaxation from the FC-region, the excited state decays on the sub-ps timescale to form the red-shifted GSI-1. Such a red-shifted species has been observed in *Syn*Cph1 and oatPhyA [27,28,45,47,68] and was assigned to the primary photoproduct state Lumi-F [47]. Our model expands this picture by attributing GSI-1 to the hot Lumi-F (~750 nm), while GSI-2 to the relaxed Lumi-F (~730 nm). The kinetics of the transition between the two Lumi-Fs and the accompanying recovery of the GSB is not purely exponential as indicated by the lifetime distribution analysis, which is expected given the short timescale and the significant vibrational energy present shortly after the excited state relaxation. The following blue shift dynamics (GSIs-2/3/4) is associated with slow adaptation/relaxation of the isomerized chromophore and its binding pocket, and ultimately leads to the formation of Meta-F (GSI-5) on the sub-ns timescale. The blue shift dynamics resembles the dynamics reported for oatPhyA P_fr_ [27,28]. In contrast to *Syn*Cph1 [45,47], in *Syn*Cph2 we do not find an indication for distinctly heterogeneous excited state kinetics.

## 3. Materials and Methods

### 3.1. Protein Preparation

Preparation of *Syn*Cph2 was described elsewhere [13]. The final buffer conditions for the experiments were set to 50 mM TRIS, pH 8.0, 300 mM NaCl, and 5 mM EDTA.

### 3.2. Stationary Characterization

UV/vis stationary absorption spectra of *Syn*Cph2 were recorded using a Specord S600 absorption spectrometer (Analytik Jena). The sample was converted into the P_r_ and the P_fr_ state by illumination with an appropriate LED (730 nm and 590 nm respectively, 1W, Thorlabs). The stationary FTIR spectra were measured on a Bruker Tensor 27 equipped with a MCT detector under the same illumination conditions as used for the UV/vis absorption measurements. The sample chamber was purged with nitrogen.

### 3.3. Vis Pump-Probe Transient Absorption Experiments

The time-resolved TA measurements were conducted using a home-built pump-probe setup, as described in detail elsewhere [69]. In short, the fundamental laser pulses (1 mJ, 775 nm, 130 fs, 1 kHz) were provided by a Ti:Sa amplifier system (Clark, MXR-CPA-iSeries). The pump pulses were generated using a home-built two stage NOPA (noncollinear optical parametric amplifier) [70,71] and were compressed in a prism compressor located between the two NOPA stages. White light continuum pulses (300–750 nm) were generated by focusing the laser fundamental beam into a CaF_2_-crystal (5 mm). These probe pulses were split into probe and reference beam. The reference beam was guided directly into a spectrograph, while the probe beam was focused at the sample position and then collected and directed into a second spectrograph. The spectrographs (AMKO Multimode) contained gratings with 600 grooves/mm blazed at 500 nm and a photodiode array with a detection range set to 400–750 nm. The instrument response function (IRF) of ~80 fs in the experiments was estimated from the pump probe cross correlation. To eliminate anisotropic contributions, the measurements were carried out under magic angle conditions (54.7° pump-probe polarization difference). The sample (OD ~0.4 at the excitation wavelength) was held in a fused silica cuvette with an optical path length of 1 mm which was constantly moved in the plane perpendicular to the direction of probe pulse propagation to avoid accumulation of photoproducts. To keep the sample in a defined state the cuvette was constantly illuminated with a high-power LED at 730 nm for the P_r_ and 590 nm for the P_fr_ state experiments.

### 3.4. Vis Pump-IR Probe Transient Absorption Experiments

The TA IR measurements were performed with a Ti:Sa regenerative amplifier (4.5 mJ, 800 nm, 90 fs, 1 kHz; Mira Legend Elite HE, Coherent, Santa Clara, CA, USA) that is used to pump two home-built collinear OPAs. The signal and idler beams of one OPA were used to generate broadband IR probe and reference pulses via difference frequency generation (DFG) in AgGaS_2_. The signal beam of the second OPA was frequency-doubled in a BBO to generate the pump pulses at 642 nm and 712 nm to excite the P_r_ and P_fr_ forms, respectively. Each form was accumulated using the same background illumination as used in the vis TA measurements described above. The relative polarization between the pump and probe/reference beams was set to magic angle. The probe and reference beams were dispersed using a 150 lines/mm grating inside a single spectrometer (Triax, Jobin Yvon), resulting in a spectral resolution of about 5 cm^−1^. The C=C and C=O spectral regions were probed in two separate spectral windows by shifting the center wavelength from 6220 nm to 5800 nm. The excitation density for the experiment in the C=O region of the P_r_ state was 1 µJ, while for the rest of the measurements 2 µJ excitation was used. The detector consisted of a 2 × 32 pixel mercury cadmium telluride (MCT) detector (Infrared Associates, USA). The IRF was about 200 fs and the pump-probe delay was mechanically scanned from −30 ps up to about 3.5 ns. The sample (OD ~0.2–0.3 at the excitation wavelength) was sandwiched between two CaF_2_ windows, separated by a 50 µm PTFE spacer [72], and continuously moved with a Lissajous scanner to ensure a fresh spot for each shot.

### 3.5. Data Analysis

Data analysis was performed using OPTIMUS (www.optimusfit.org, accessed on 23 August 2021) [52]. We used the model independent lifetime distribution analysis (LDA) for the ultrafast TA data. This method can account for non-exponential kinetics. In LDA, a set of 100 exponential functions with fixed lifetimes equally distributed on a log10 scale is used to describe the data by determining the pre-exponential amplitudes. Those amplitudes can then be displayed for each detection wavelength in form of a contour plot called lifetime density map (LDM) [73]. LDMs are similar to decay-associated spectra: Positive amplitudes account for decay of excited state and product absorption (ESA, PA) or rise of ground state bleach or stimulated emission (GSB and SE). Negative amplitudes correspond to the rise of absorption (ESA, PA) or the decay of GSB and SE.

In addition, we performed global target analysis [52,57]. In short, kinetic models are fitted directly to the experimental data to verify their adequacy. The analysis results in the so-called species-associated difference spectra (SADS), or when a simple sequential model (A→B→C…) is used in evolution-associated difference spectra (EADS). Unlike decay-associated difference spectra (DADS) from global lifetime analysis, SADS and EADS contain pure spectral information—SADS represent the spectra of the species in the kinetic model, while EADS show the overall spectral evolution. Global target analysis also delivers the kinetic rates of the different reaction steps, as well as the lifetimes and the conventional DADS. Our implementation allows us to constrain the quantum yield for formation of the last observable (on the experiment timescale) photoproduct state. This limits the parameter fitting space and facilitates the determination of an adequate model. Since this type of analysis involves a certain level of over-parametrization, the solutions are not unique. Therefore, the adequacy of the models that fit the experimental data is judged based on whether the SADS and the kinetic rates are physically reasonable.

## 4. Conclusions

Our work provides insight into the unexplored ultrafast dynamics of knotless phytochromes. We show that the excited state P_r_ dynamics of *Syn*Cph2 occur on the timescale of hundreds of picoseconds, similarly to All2699g1g2 [50], bacteriophytochromes [35,36,56], and a number of red/green CBCRs [40,41,42,43]. In contrast to the P_r_ forms of some canonical phytochromes like *Syn*Cph1, here we do not find distinctly heterogeneous but rather distributed kinetics, which we attribute to reorganization of the chromophore-binding pocket that controls the isomerization dynamics. The P_fr_ dynamics of *Syn*Cph2 resembles the one observed in oatPhyA with a fast, sub-ps excited state decay followed by consecutive blue shifts of the formed GSIs. In general, the dynamics is also very similar to what is reported in *Syn*Cph1. However, in *Syn*Cph2 the data again do not point to spectroscopically separable ground states.

Quite remarkably, both the forward and the reverse dynamics of the knotless phytochrome *Syn*Cph2, which lacks the PAS domain, can be readily compared to other phytochromes containing a PAS-GAF-PHY array as their PSM. Furthermore, the P_r_ → P_fr_ dynamics of *Syn*Cph2 is also very similar to some CBCRs which are composed only of a single GAF domain. Therefore, it emerges that despite the great variation in the relevant structural components (domains and composition), the primary photoconversion mechanism appears to be fundamentally conserved in most phytochromes. Consequently, the key to the detailed understanding of phytochrome photochemistry is the identification of common structural patterns regulating and defining the photoisomerization step. Importantly, this will also result in a general design principle for optimized phytochromes for biotechnological applications.

## Data Availability

Data is available from the authors upon reasonable request.

## Supplementary Materials

The following are available online at https://www.mdpi.com/article/10.3390/ijms221910690/s1, Figure S1/S2: Transients and difference spectra at selected delay times for the visible TA data of *Syn*Cph2 for the P_r_ → P_fr_ and P_fr_ → P_r_ dynamics respectively, Figure S3: FTIR difference spectrum of Cph2, Figures S4–S6: Additional kinetic models used to fit the TA data of the Pfr dynamics of *Syn*Cph2, Figure S7: Comparison of data and fit for the ultrafast reverse dynamics of Cph2 using the kinetic model displayed in Figure 7 for selected wavelengths.
