# Supplementary material for: Ultrafast Photoconversion Dynamics of the Knotless Phytochrome SynCph2
Source: Int J Mol Sci. 2021 Oct 2;22(19):10690. doi: 10.3390/ijms221910690 (PMC8508867; doi:10.3390/ijms221910690)
Supplement: Supplementary file 1 [file ijms-22-10690-s001.zip › ijms-1366260-supplementary.pdf]

Supporting Information

Ultrafast photoconversion dynamics of the knotless phytochrome *Syn*Cph2

Tobias Fischer [1], Luuk J. G. W. van Wilderen [2], Petra Gnau [3], Jens Bredenbeck [2], Lars-Oliver Essen[3,4] and Josef Wachtveitl[1,*] Chavdar Slavov[1,*]

[1] Institute of Physical and Theoretical Chemistry, Goethe University Frankfurt am Main, Max-von-Laue Straße 7, 60438 Frankfurt, Germany, fischer@theochem.uni-frankfurt.de (T.F.); wveitl@theochem.uni-frankfurt.de (J.W.); chslavov@theochem.uni-frankfurt.de (C.S.)

[2] Institute of Biophysics, Goethe University Frankfurt am Main, Max-von-Laue Straße 1, 60438 Frankfurt, Germany; vanwilderen@biophysik.uni-frankfurt.de (L.J.G.W.v.W.); bredenbeck@biophysik.uni-frankfurt.de (J.B.)

[3] Department of Chemistry, Philipps-Universität Marburg, Hans-Meerwein-Straße 4, 35032 Marburg, Germany; gnau@staff.uni-marburg.de (P.G.); essen@chemie.uni-marburg.de (L.-O.E.)

[4] Center for Synthetic Microbiology, Philipps-Universität Marburg, Hans-Meerwein-Straße 6, 35032 Marburg, Germany), essen@chemie.uni-marburg.de

* Correspondence: chslavov@theochem.uni-frankfurt.de (C.S.); wveitl@theochem.uni-frankfurt.de (J.W.)

Abstract

The family of phytochrome photoreceptors contains proteins with different domain architectures and spectral properties. Knotless phytochromes are one of the three main subgroups classified by their distinct lack of the PAS domain in their photosensory core module, which is in contrast to the canonical PAS-GAF-PHY array. Despite intensive research on the ultrafast photodynamics of phytochromes, little is known about the primary kinetics in knotless phytochromes. Here, we present the ultrafast $P_r \leftrightarrows P_{fr}$ photodynamics of *Syn*Cph2, the best-known knotless phytochrome. Our results show that the excited state lifetime of P_r* (~200 ps) is similar to bacteriophytochromes, but much longer than in most canonical phytochromes. We assign the slow P_r* kinetics to relaxation of the chromophore-binding pocket that controls the bilin chromophore's isomerization step. The P_{fr} photoconversion dynamics starts with a faster excited state relaxation than in canonical phytochromes, but, despite the differences in the respective domain architectures, proceeds via similar ground state intermediate steps up to Meta-F. Based on our observations, we propose that the kinetic features and overall dynamics of the ultrafast photoreaction are determined to a great extent by the geometrical circumstances (i.e. available space and flexibility) within the binding pocket, while the general reaction steps following the photoexcitation are most likely conserved among the red/far-red phytochromes.

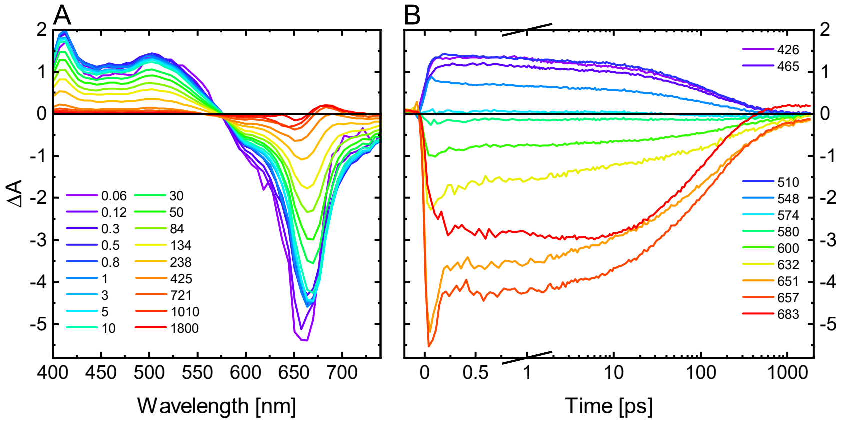

Figure S1. TA data from the primary forward dynamics ($P_r \rightarrow P_{fr}$) of SynCph2. A) Difference spectra at selected delay times (in ps) and B) transient absorption changes at selected detection wavelengths (in nm). Positive signals correspond to excited state (ESA) and product absorption (PA), while negative signals correspond to ground state bleach (GSB) and stimulated emission (SE).

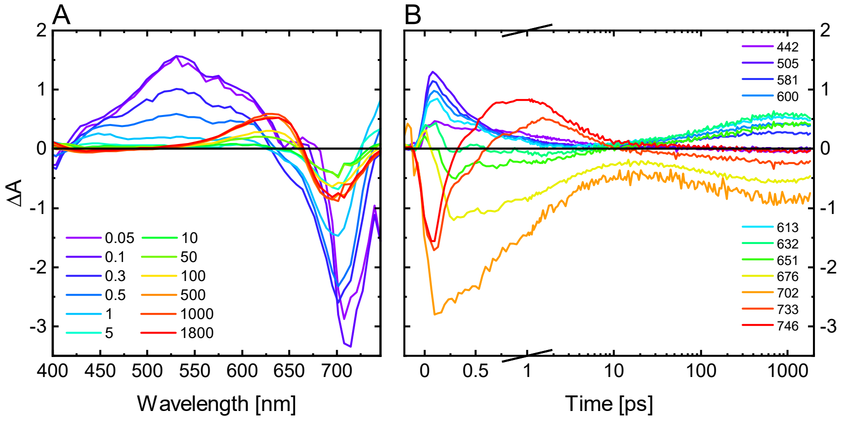

Figure S2. TA data from the primary reverse dynamics ($P_{fr} \rightarrow P_r$) of SynCph2. A) Difference spectra at selected delay times (in ps) and B) transient absorption changes at selected detection wavelengths (in nm). Positive signals correspond to excited state (ESA) and product absorption (PA), while negative signals correspond to ground state bleach (GSB) and stimulated emission (SE).

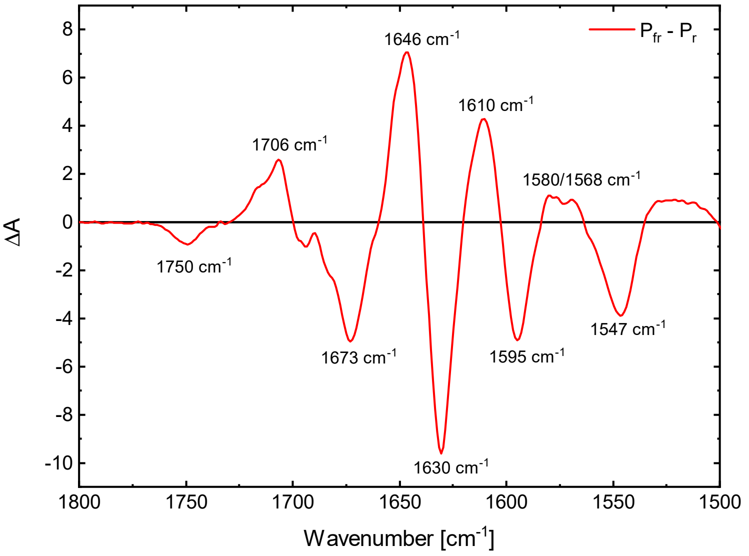

Figure S3. FTIR difference spectrum of Cph2. Positive contributions correspond to the P_{fr} state while negative contributions can be assigned to the P_r state.

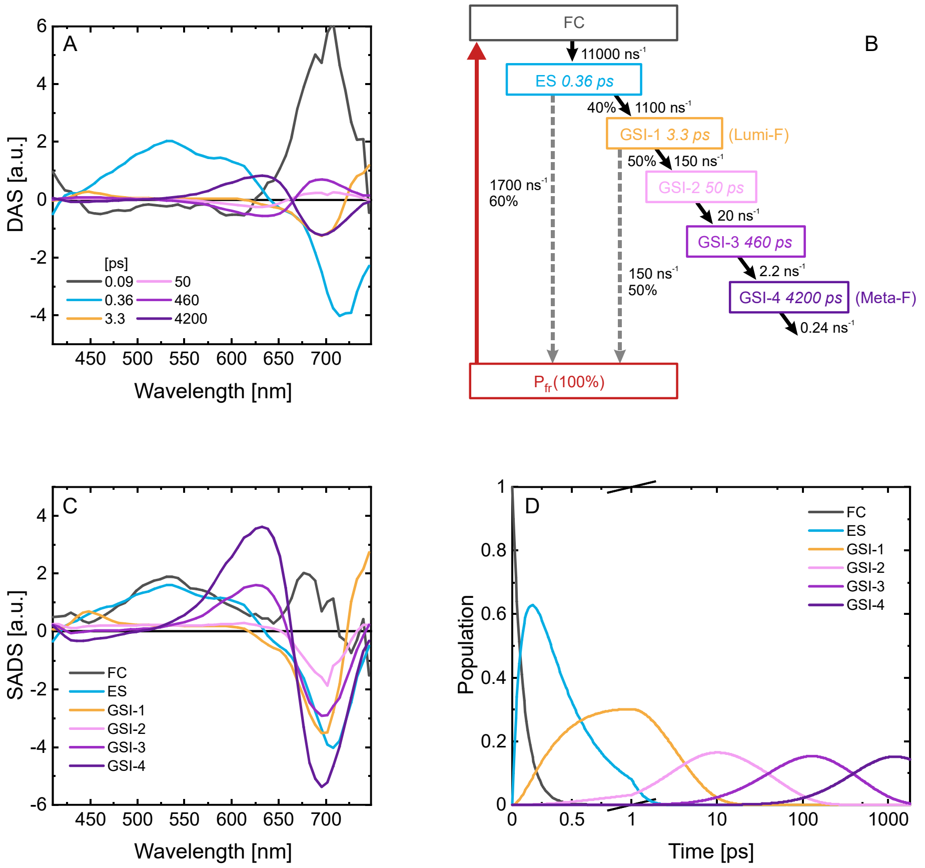

Figure S4. DAS (A), SADS (C) and populations (D) of individual states obtained from the GTA on the transient absorption data of P_{fr}. The assignment to excited state or GS dynamics is based on the presence of ESA in the 410 to 600 nm range. B) Schematic representation of the kinetic model showing the rates (in ns^{-1}) and the decay lifetimes (in ps). Colors match the respective DAS, SADS and populations. The overall QY of the productive pathway was set to 0.2.*

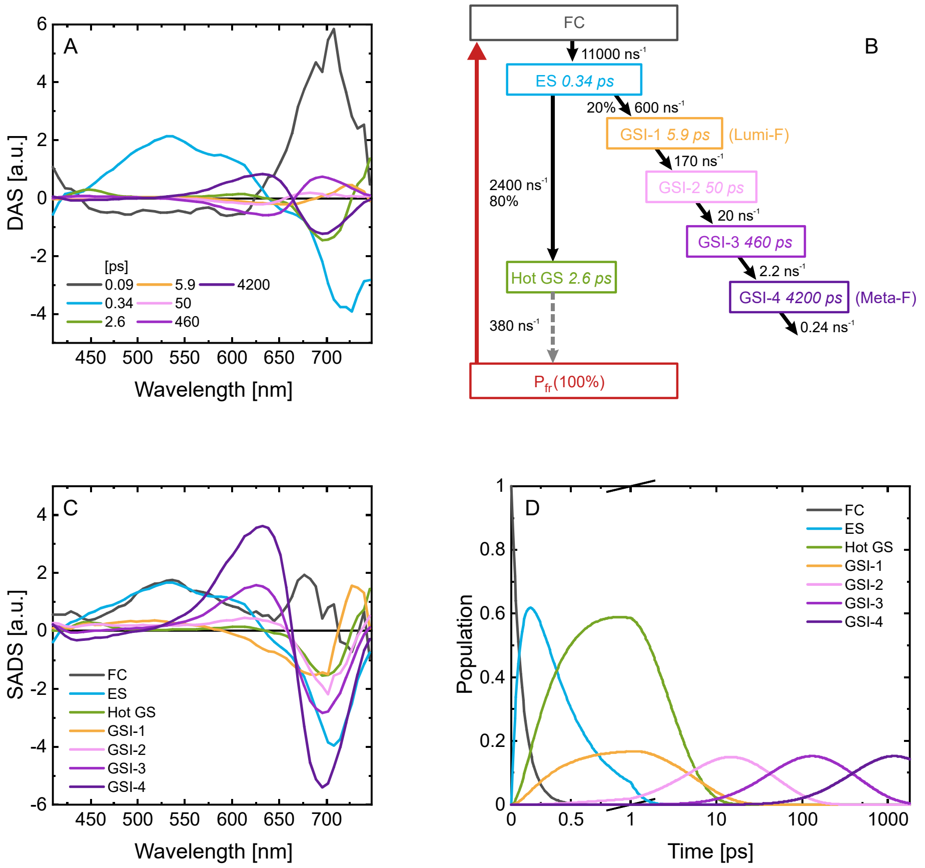

Figure S5. DAS (A), SADS (C) and populations (D) of individual states obtained from the GTA on the transient absorption data of P_{fr}. The assignment to excited state or GS dynamics is based on the presence of ESA in the 410 to 600 nm range. B) Schematic representation of the kinetic model showing the rates (in ns^{-1}) and the decay lifetimes (in ps). Colors match the respective DAS, SADS and populations. The overall QY of the productive pathway was set to 0.2.*

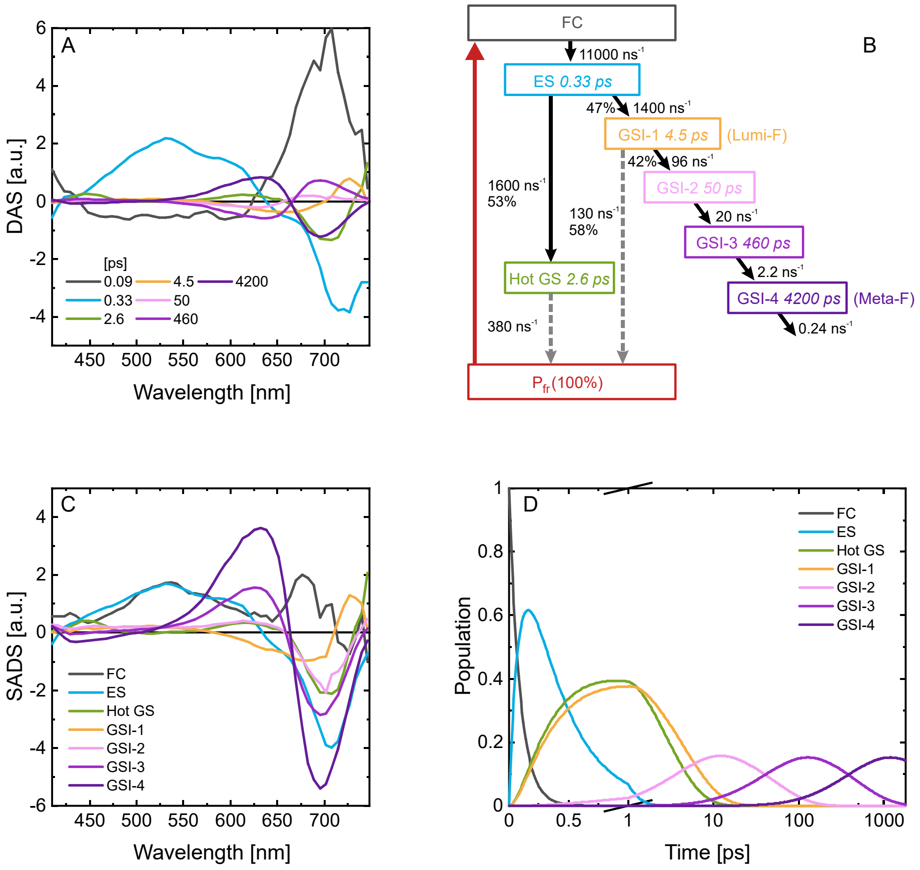

Figure S6. DAS (A), SADS (C) and populations (D) of individual states obtained from the GTA on the transient absorption data of P_{fr}. The assignment to excited state or GS dynamics is based on the presence of ESA in the 410 to 600 nm range. B) Schematic representation of the kinetic model showing the rates (in ns^{-1} a) and the decay lifetimes (in ps). Colors match the respective DAS, SADS and populations. The overall QY of the productive pathways was set to 0.2.*

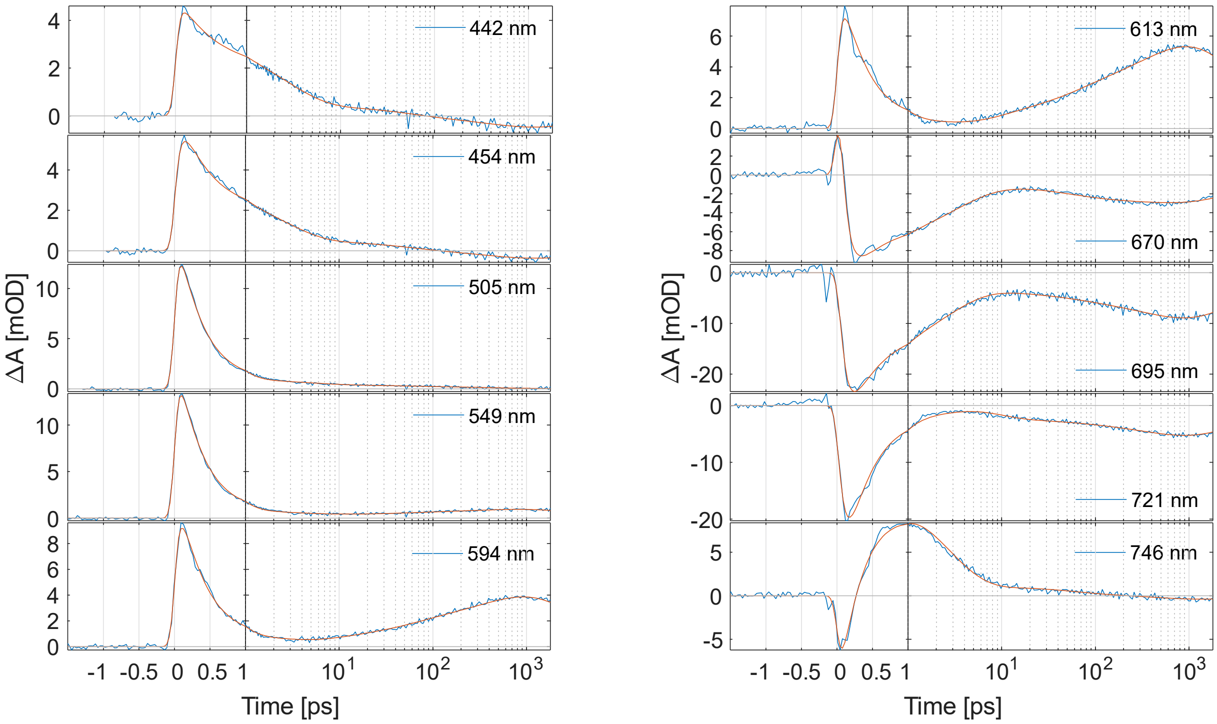

Figure S7. Comparison of data and fit for transients at selected wavelengths from the primary reverse dynamics ($P_{fr} \longrightarrow P_r$) of SynCph2. The data are displayed in blue while the fit using the kinetic model in Fig. 7 is displayed in orange.
